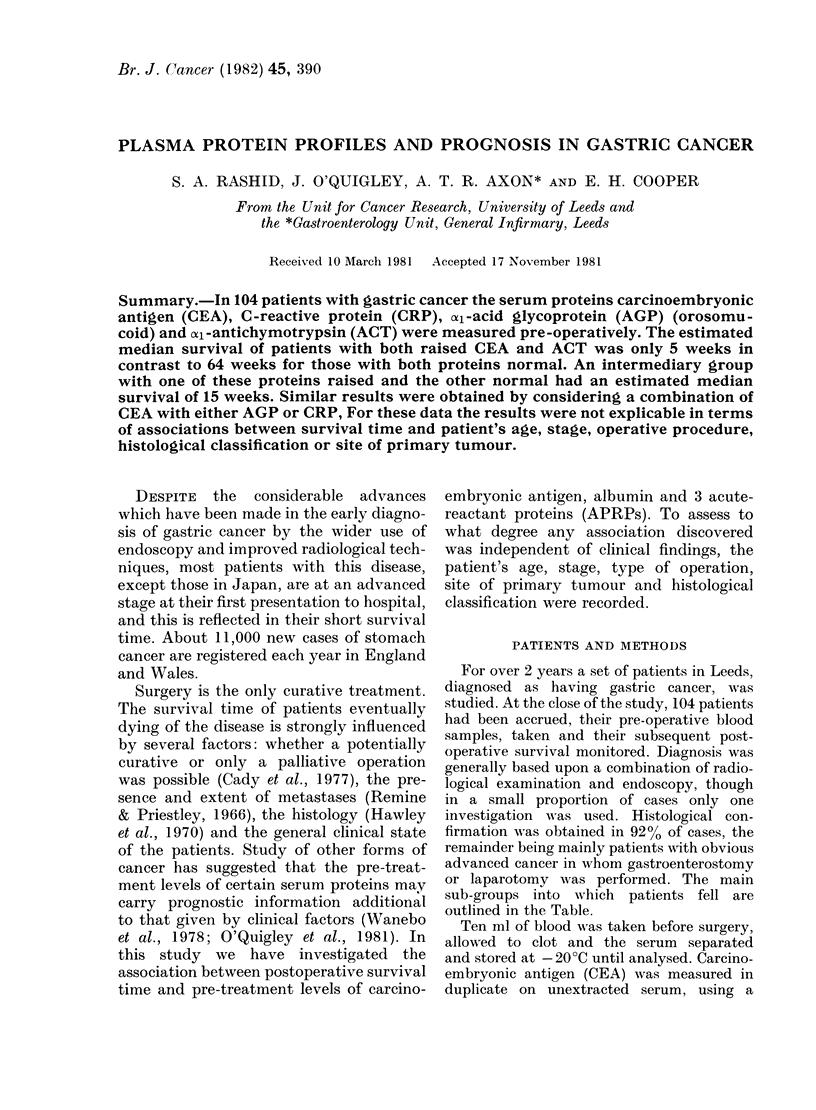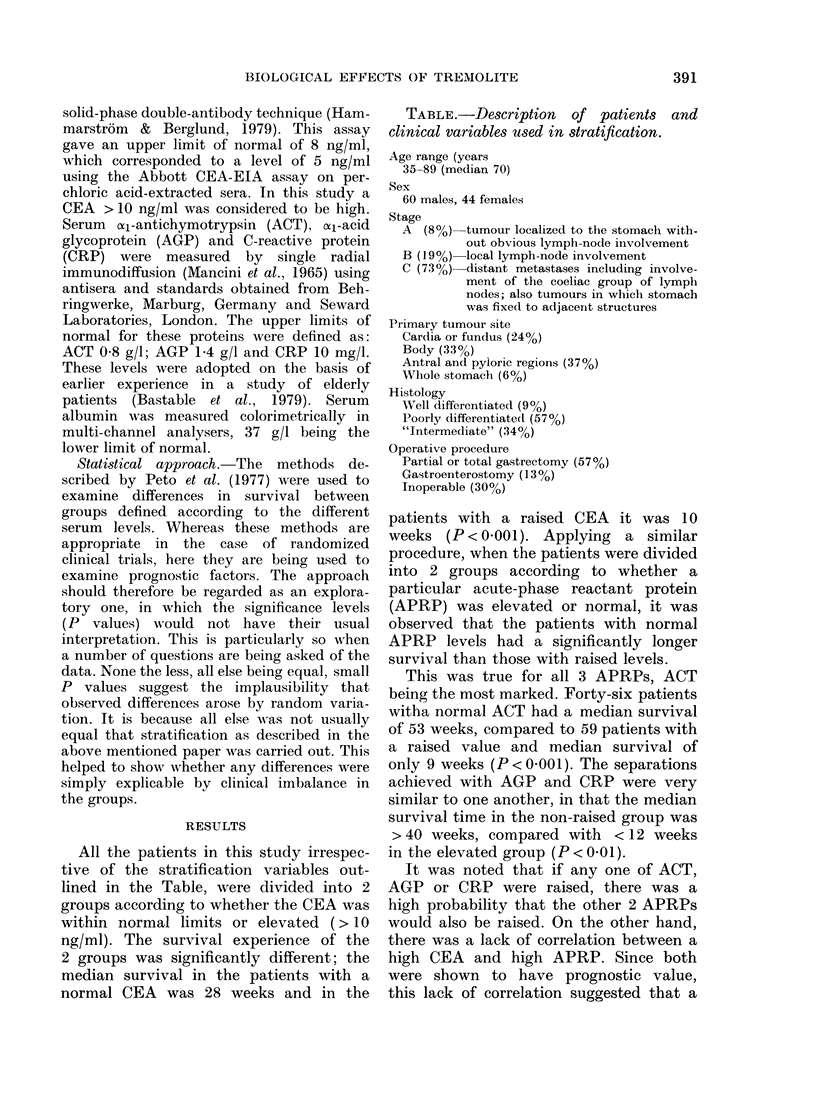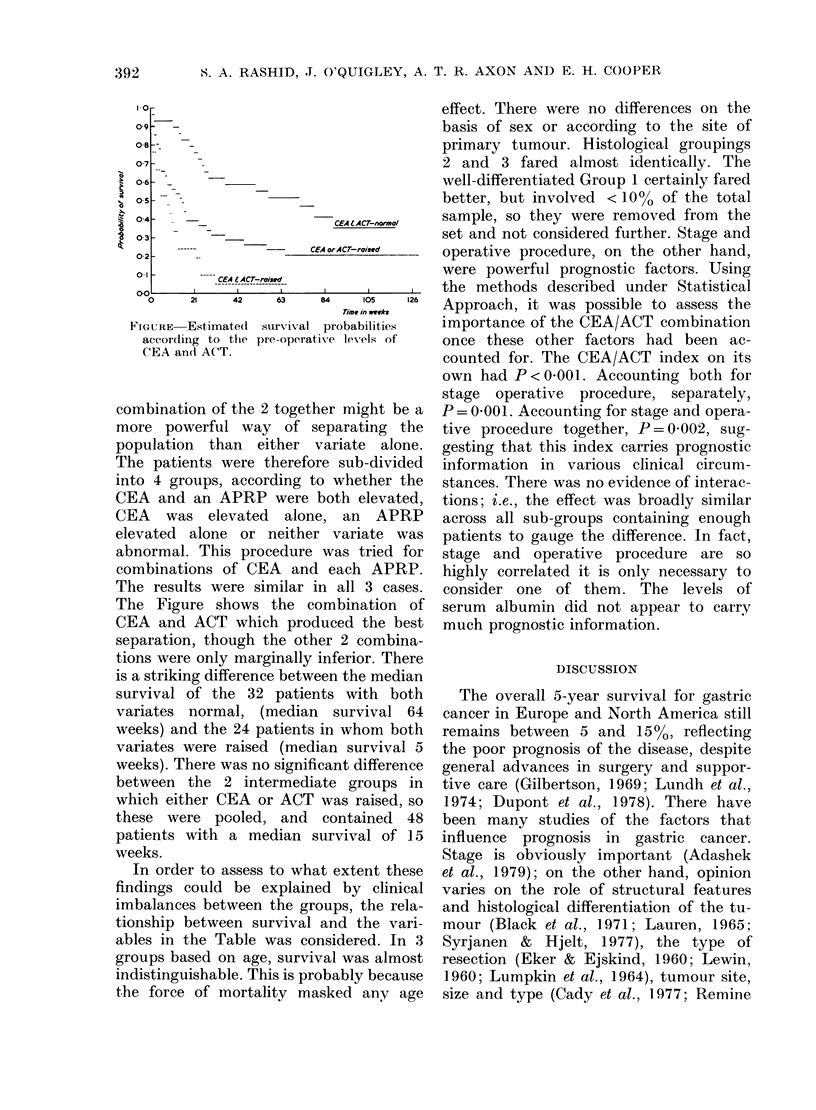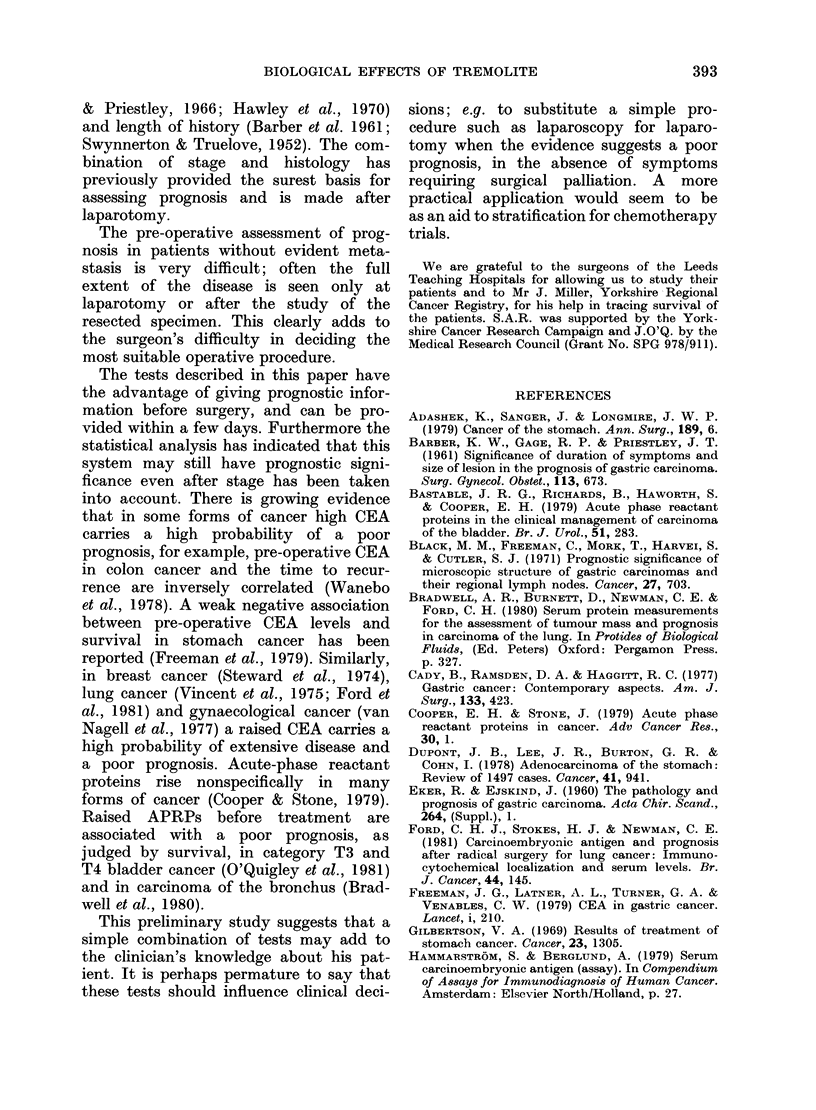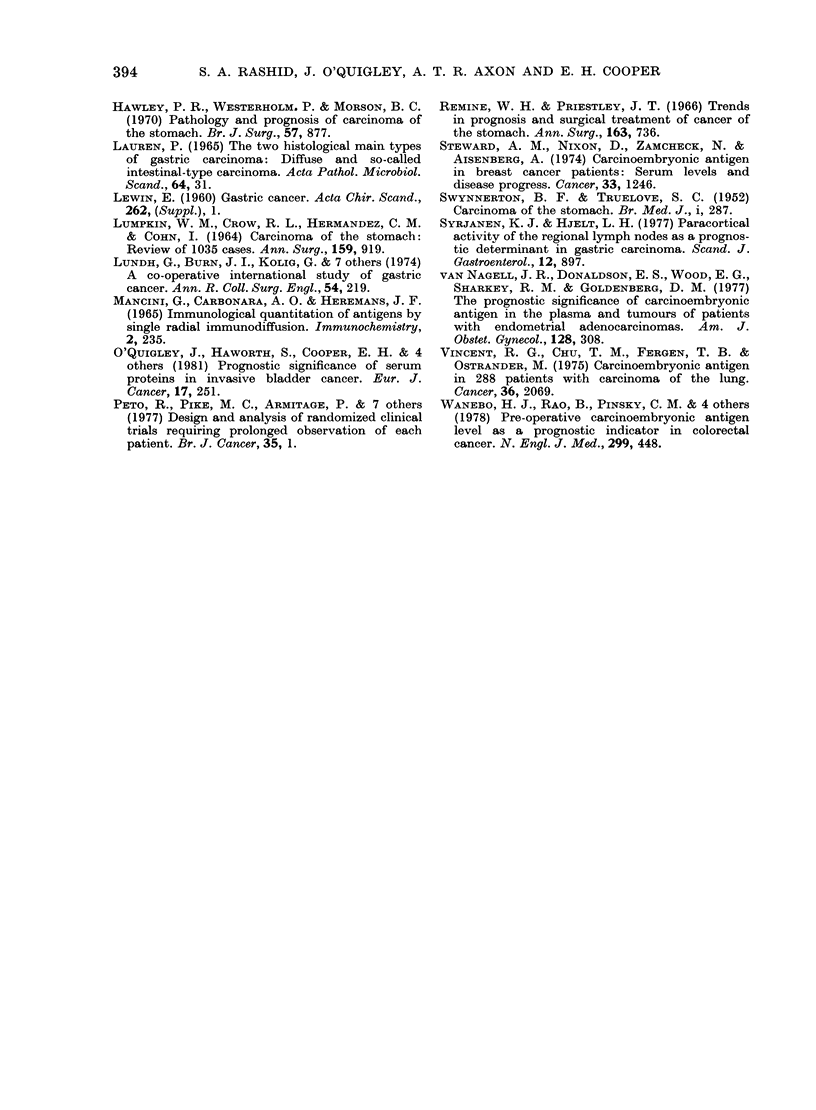# Plasma protein profiles and prognosis in gastric cancer.

**DOI:** 10.1038/bjc.1982.66

**Published:** 1982-03

**Authors:** S. A. Rashid, J. O'Quigley, A. T. Axon, E. H. Cooper

## Abstract

In 104 patients with gastric cancer the serum proteins carcinoembryonic antigen (CEA), C-reactive protein (CRP), alpha 1-acid glycoprotein (AGP) (orosomucoid) and alpha 1-antichymotrypsin (ACT) were measured pre-operatively. The estimated median survival of patients with both raised CEA and ACT was only 5 weeks in contrast to 64 weeks for those with both proteins normal. An intermediary group with one of these proteins raised and the other normal had an estimated median survival of 15 weeks. Similar results were obtained by considering a combination of CEA with either AGP or CRP. For these data the results were not explicable in terms of associations between survival time and patient's age, stage, operative procedure, histological classification or site of primary tumour.


Br. J. Cancer (1982) 45, 390

### PLASMA PROTEIN PROFILES AND PROGNOSIS IN GASTRIC CANCER

S. A. RASHID, J. O'QUIGLEY, A. T. R. AXON* AND E. H. COOPER

*From the Unit for Cancer Research, University of Leeds and the *Gastroenterology Unit, General Infirmary, Leeds*

Received 10 March 1981 Accepted 17 November 1981

**Summary.—In 104 patients with gastric cancer the serum proteins carcinoembryonic antigen (CEA), C-reactive protein (CRP), $\alpha_1$-acid glycoprotein (AGP) (orosomucoid) and $\alpha_1$-antichymotrypsin (ACT) were measured pre-operatively. The estimated median survival of patients with both raised CEA and ACT was only 5 weeks in contrast to 64 weeks for those with both proteins normal. An intermediary group with one of these proteins raised and the other normal had an estimated median survival of 15 weeks. Similar results were obtained by considering a combination of CEA with either AGP or CRP, For these data the results were not explicable in terms of associations between survival time and patient's age, stage, operative procedure, histological classification or site of primary tumour.**

DESPITE the considerable advances which have been made in the early diagnosis of gastric cancer by the wider use of endoscopy and improved radiological techniques, most patients with this disease, except those in Japan, are at an advanced stage at their first presentation to hospital, and this is reflected in their short survival time. About 11,000 new cases of stomach cancer are registered each year in England and Wales.

Surgery is the only curative treatment. The survival time of patients eventually dying of the disease is strongly influenced by several factors: whether a potentially curative or only a palliative operation was possible (Cady *et al.*, 1977), the presence and extent of metastases (Remine & Priestley, 1966), the histology (Hawley *et al.*, 1970) and the general clinical state of the patients. Study of other forms of cancer has suggested that the pre-treatment levels of certain serum proteins may carry prognostic information additional to that given by clinical factors (Wanebo *et al.*, 1978; O'Quigley *et al.*, 1981). In this study we have investigated the association between postoperative survival time and pre-treatment levels of carcinoembryonic antigen, albumin and 3 acute-reactant proteins (APRPs). To assess to what degree any association discovered was independent of clinical findings, the patient's age, stage, type of operation, site of primary tumour and histological classification were recorded.

### PATIENTS AND METHODS

For over 2 years a set of patients in Leeds, diagnosed as having gastric cancer, was studied. At the close of the study, 104 patients had been accrued, their pre-operative blood samples, taken and their subsequent post-operative survival monitored. Diagnosis was generally based upon a combination of radiological examination and endoscopy, though in a small proportion of cases only one investigation was used. Histological confirmation was obtained in 92% of cases, the remainder being mainly patients with obvious advanced cancer in whom gastroenterostomy or laparotomy was performed. The main sub-groups into which patients fell are outlined in the Table.

Ten ml of blood was taken before surgery, allowed to clot and the serum separated and stored at −20°C until analysed. Carcinoembryonic antigen (CEA) was measured in duplicate on unextracted serum, using a solid-phase double-antibody technique (Hammarström & Berglund, 1979). This assay gave an upper limit of normal of 8 ng/ml, which corresponded to a level of 5 ng/ml using the Abbott CEA-EIA assay on perchloric acid-extracted sera. In this study a CEA $>10$ ng/ml was considered to be high. Serum $\alpha_1$-antichymotrypsin (ACT), $\alpha_1$-acid glycoprotein (AGP) and C-reactive protein (CRP) were measured by single radial immunodiffusion (Mancini *et al.*, 1965) using antisera and standards obtained from Behringwerke, Marburg, Germany and Seward Laboratories, London. The upper limits of normal for these proteins were defined as: ACT 0·8 g/l; AGP 1·4 g/l and CRP 10 mg/l. These levels were adopted on the basis of earlier experience in a study of elderly patients (Bastable *et al.*, 1979). Serum albumin was measured colorimetrically in multi-channel analysers, 37 g/l being the lower limit of normal.

*Statistical approach.*—The methods described by Peto *et al.* (1977) were used to examine differences in survival between groups defined according to the different serum levels. Whereas these methods are appropriate in the case of randomized clinical trials, here they are being used to examine prognostic factors. The approach should therefore be regarded as an exploratory one, in which the significance levels ($P$ values) would not have their usual interpretation. This is particularly so when a number of questions are being asked of the data. None the less, all else being equal, small $P$ values suggest the implausibility that observed differences arose by random variation. It is because all else was not usually equal that stratification as described in the above mentioned paper was carried out. This helped to show whether any differences were simply explicable by clinical imbalance in the groups.

### RESULTS

All the patients in this study irrespective of the stratification variables outlined in the Table, were divided into 2 groups according to whether the CEA was within normal limits or elevated ($>10$ ng/ml). The survival experience of the 2 groups was significantly different; the median survival in the patients with a normal CEA was 28 weeks and in the patients with a raised CEA it was 10 weeks ($P<0{\cdot}001$). Applying a similar procedure, when the patients were divided into 2 groups according to whether a particular acute-phase reactant protein (APRP) was elevated or normal, it was observed that the patients with normal APRP levels had a significantly longer survival than those with raised levels.

This was true for all 3 APRPs, ACT being the most marked. Forty-six patients witha normal ACT had a median survival of 53 weeks, compared to 59 patients with a raised value and median survival of only 9 weeks ($P<0{\cdot}001$). The separations achieved with AGP and CRP were very similar to one another, in that the median survival time in the non-raised group was $>40$ weeks, compared with $<12$ weeks in the elevated group ($P<0{\cdot}01$).

It was noted that if any one of ACT, AGP or CRP were raised, there was a high probability that the other 2 APRPs would also be raised. On the other hand, there was a lack of correlation between a high CEA and high APRP. Since both were shown to have prognostic value, this lack of correlation suggested that a

TABLE.—*Description of patients and clinical variables used in stratification.*

Age range (years
 35–89 (median 70)

Sex
 60 males, 44 females

Stage
 A (8%)—tumour localized to the stomach without obvious lymph-node involvement
 B (19%)—local lymph-node involvement
 C (73%)—distant metastases including involvement of the coeliac group of lymph nodes; also tumours in which stomach was fixed to adjacent structures

Primary tumour site
 Cardia or fundus (24%)
 Body (33%)
 Antral and pyloric regions (37%)
 Whole stomach (6%)

Histology

 Well differentiated (9%)

 Poorly differentiated (57%)

 "Intermediate" (34%)

Operative procedure
 Partial or total gastrectomy (57%)
 Gastroenterostomy (13%)
 Inoperable (30%)

FIGURE—Estimated survival probabilities according to the pre-operative levels of CEA and ACT.

combination of the 2 together might be a more powerful way of separating the population than either variate alone. The patients were therefore sub-divided into 4 groups, according to whether the CEA and an APRP were both elevated, CEA was elevated alone, an APRP elevated alone or neither variate was abnormal. This procedure was tried for combinations of CEA and each APRP. The results were similar in all 3 cases. The Figure shows the combination of CEA and ACT which produced the best separation, though the other 2 combinations were only marginally inferior. There is a striking difference between the median survival of the 32 patients with both variates normal, (median survival 64 weeks) and the 24 patients in whom both variates were raised (median survival 5 weeks). There was no significant difference between the 2 intermediate groups in which either CEA or ACT was raised, so these were pooled, and contained 48 patients with a median survival of 15 weeks.

In order to assess to what extent these findings could be explained by clinical imbalances between the groups, the relationship between survival and the variables in the Table was considered. In 3 groups based on age, survival was almost indistinguishable. This is probably because the force of mortality masked any age effect. There were no differences on the basis of sex or according to the site of primary tumour. Histological groupings 2 and 3 fared almost identically. The well-differentiated Group 1 certainly fared better, but involved $<10\%$ of the total sample, so they were removed from the set and not considered further. Stage and operative procedure, on the other hand, were powerful prognostic factors. Using the methods described under Statistical Approach, it was possible to assess the importance of the CEA/ACT combination once these other factors had been accounted for. The CEA/ACT index on its own had $P<0{\cdot}001$. Accounting both for stage operative procedure, separately, $P=0{\cdot}001$. Accounting for stage and operative procedure together, $P=0{\cdot}002$, suggesting that this index carries prognostic information in various clinical circumstances. There was no evidence of interactions; *i.e.*, the effect was broadly similar across all sub-groups containing enough patients to gauge the difference. In fact, stage and operative procedure are so highly correlated it is only necessary to consider one of them. The levels of serum albumin did not appear to carry much prognostic information.

### DISCUSSION

The overall 5-year survival for gastric cancer in Europe and North America still remains between 5 and 15%, reflecting the poor prognosis of the disease, despite general advances in surgery and supportive care (Gilbertson, 1969; Lundh *et al.*, 1974; Dupont *et al.*, 1978). There have been many studies of the factors that influence prognosis in gastric cancer. Stage is obviously important (Adashek *et al.*, 1979); on the other hand, opinion varies on the role of structural features and histological differentiation of the tumour (Black *et al.*, 1971; Lauren, 1965; Syrjanen & Hjelt, 1977), the type of resection (Eker & Ejskind, 1960; Lewin, 1960; Lumpkin *et al.*, 1964), tumour site, size and type (Cady *et al.*, 1977; Remine

& Priestley, 1966; Hawley *et al.*, 1970) and length of history (Barber *et al.* 1961; Swynnerton & Truelove, 1952). The combination of stage and histology has previously provided the surest basis for assessing prognosis and is made after laparotomy.

The pre-operative assessment of prognosis in patients without evident metastasis is very difficult; often the full extent of the disease is seen only at laparotomy or after the study of the resected specimen. This clearly adds to the surgeon's difficulty in deciding the most suitable operative procedure.

The tests described in this paper have the advantage of giving prognostic information before surgery, and can be provided within a few days. Furthermore the statistical analysis has indicated that this system may still have prognostic significance even after stage has been taken into account. There is growing evidence that in some forms of cancer high CEA carries a high probability of a poor prognosis, for example, pre-operative CEA in colon cancer and the time to recurrence are inversely correlated (Wanebo *et al.*, 1978). A weak negative association between pre-operative CEA levels and survival in stomach cancer has been reported (Freeman *et al.*, 1979). Similarly, in breast cancer (Steward *et al.*, 1974), lung cancer (Vincent *et al.*, 1975; Ford *et al.*, 1981) and gynaecological cancer (van Nagell *et al.*, 1977) a raised CEA carries a high probability of extensive disease and a poor prognosis. Acute-phase reactant proteins rise nonspecifically in many forms of cancer (Cooper & Stone, 1979). Raised APRPs before treatment are associated with a poor prognosis, as judged by survival, in category T3 and T4 bladder cancer (O'Quigley *et al.*, 1981) and in carcinoma of the bronchus (Bradwell *et al.*, 1980).

This preliminary study suggests that a simple combination of tests may add to the clinician's knowledge about his patient. It is perhaps permature to say that these tests should influence clinical decisions; *e.g.* to substitute a simple procedure such as laparoscopy for laparotomy when the evidence suggests a poor prognosis, in the absence of symptoms requiring surgical palliation. A more practical application would seem to be as an aid to stratification for chemotherapy trials.

We are grateful to the surgeons of the Leeds Teaching Hospitals for allowing us to study their patients and to Mr J. Miller, Yorkshire Regional Cancer Registry, for his help in tracing survival of the patients. S.A.R. was supported by the Yorkshire Cancer Research Campaign and J.O'Q. by the Medical Research Council (Grant No. SPG 978/911).

### REFERENCES

ADASHEK, K., SANGER, J. & LONGMIRE, J. W. P. (1979) Cancer of the stomach. *Ann. Surg.*, **189**, 6.

BARBER, K. W., GAGE, R. P. & PRIESTLEY, J. T. (1961) Significance of duration of symptoms and size of lesion in the prognosis of gastric carcinoma. *Surg. Gynecol. Obstet.*, **113**, 673.

BASTABLE, J. R. G., RICHARDS, B., HAWORTH, S. & COOPER, E. H. (1979) Acute phase reactant proteins in the clinical management of carcinoma of the bladder. *Br. J. Urol.*, **51**, 283.

BLACK, M. M., FREEMAN, C., MORK, T., HARVEI, S. & CUTLER, S. J. (1971) Prognostic significance of microscopic structure of gastric carcinomas and their regional lymph nodes. *Cancer*, **27**, 703.

BRADWELL, A. R., BURNETT, D., NEWMAN, C. E. & FORD, C. H. (1980) Serum protein measurements for the assessment of tumour mass and prognosis in carcinoma of the lung. In *Protides of Biological Fluids*, (Ed. Peters) Oxford: Pergamon Press. p. 327.

CADY, B., RAMSDEN, D. A. & HAGGITT, R. C. (1977) Gastric cancer: Contemporary aspects. *Am. J. Surg.*, **133**, 423.

COOPER, E. H. & STONE, J. (1979) Acute phase reactant proteins in cancer. *Adv Cancer Res.*, **30**, 1.

DUPONT, J. B., LEE, J. R., BURTON, G. R. & COHN, I. (1978) Adenocarcinoma of the stomach: Review of 1497 cases. *Cancer*, **41**, 941.

EKER, R. & EJSKIND, J. (1960) The pathology and prognosis of gastric carcinoma. *Acta Chir. Scand.*, **264**, (Suppl.), 1.

FORD, C. H. J., STOKES, H. J. & NEWMAN, C. E. (1981) Carcinoembryonic antigen and prognosis after radical surgery for lung cancer: Immunocytochemical localization and serum levels. *Br. J. Cancer*, **44**, 145.

FREEMAN, J. G., LATNER, A. L., TURNER, G. A. & VENABLES, C. W. (1979) CEA in gastric cancer. *Lancet*, i, 210.

GILBERTSON, V. A. (1969) Results of treatment of stomach cancer. *Cancer*, **23**, 1305.

HAMMARSTRÖM, S. & BERGLUND, A. (1979) Serum carcinoembryonic antigen (assay). In *Compendium of Assays for Immunodiagnosis of Human Cancer*. Amsterdam: Elsevier North/Holland, p. 27.

HAWLEY, P. R., WESTERHOLM. P. & MORSON, B. C. (1970) Pathology and prognosis of carcinoma of the stomach. *Br. J. Surg.*, **57,** 877.

LAUREN, P. (1965) The two histological main types of gastric carcinoma: Diffuse and so-called intestinal-type carcinoma. *Acta Pathol. Microbiol. Scand.*, **64,** 31.

LEWIN, E. (1960) Gastric cancer. *Acta Chir. Scand.*, **262,** (*Suppl.*), 1.

LUMPKIN, W. M., CROW, R. L., HERMANDEZ, C. M. & COHN, I. (1964) Carcinoma of the stomach: Review of 1035 cases. *Ann. Surg.*, **159,** 919.

LUNDH, G., BURN, J. I., KOLIG, G. & 7 others (1974) A co-operative international study of gastric cancer. *Ann. R. Coll. Surg. Engl.*, **54,** 219.

MANCINI, G., CARBONARA, A. O. & HEREMANS, J. F. (1965) Immunological quantitation of antigens by single radial immunodiffusion. *Immunochemistry*, **2,** 235.

O'QUIGLEY, J., HAWORTH, S., COOPER, E. H. & 4 others (1981) Prognostic significance of serum proteins in invasive bladder cancer. *Eur. J. Cancer*, **17,** 251.

PETO, R., PIKE, M. C., ARMITAGE, P. & 7 others (1977) Design and analysis of randomized clinical trials requiring prolonged observation of each patient. *Br. J. Cancer*, **35,** 1.

REMINE, W. H. & PRIESTLEY, J. T. (1966) Trends in prognosis and surgical treatment of cancer of the stomach. *Ann. Surg.*, **163,** 736.

STEWARD, A. M., NIXON, D., ZAMCHECK, N. & AISENBERG, A. (1974) Carcinoembryonic antigen in breast cancer patients: Serum levels and disease progress. *Cancer*, **33,** 1246.

SWYNNERTON, B. F. & TRUELOVE, S. C. (1952) Carcinoma of the stomach. *Br. Med. J.*, i, 287.

SYRJANEN, K. J. & HJELT, L. H. (1977) Paracortical activity of the regional lymph nodes as a prognostic determinant in gastric carcinoma. *Scand. J. Gastroenterol.*, **12,** 897.

VAN NAGELL, J. R., DONALDSON, E. S., WOOD, E. G., SHARKEY, R. M. & GOLDENBERG, D. M. (1977) The prognostic significance of carcinoembryonic antigen in the plasma and tumours of patients with endometrial adenocarcinomas. *Am. J. Obstet. Gynecol.*, **128,** 308.

VINCENT, R. G., CHU, T. M., FERGEN, T. B. & OSTRANDER, M. (1975) Carcinoembryonic antigen in 288 patients with carcinoma of the lung. *Cancer*, **36,** 2069.

WANEBO, H. J., RAO, B., PINSKY, C. M. & 4 others (1978) Pre-operative carcinoembryonic antigen level as a prognostic indicator in colorectal cancer. *N. Engl. J. Med.*, **299,** 448.